# Concomitant laparoscopic urological procedures — Does it contribute to morbidity?

**DOI:** 10.4103/0972-9941.65168

**Published:** 2010

**Authors:** Kamlesh Maurya, S E Sivanandam, Sudhir Sukumar, Sanjay Bhat, Ginil Kumar, Balagopal Nair

**Affiliations:** Department of Urology, Amrita Institute of Medical Sciences, Kochi, India

Dear Sir,

We have gone through the comments. We accept that the number of patients in our study is low, as concomitant surgery is not a standard norm. The cases we have done are highly selected based on indications and co-morbidities. In many cases that had contraindications, we preferred to do open surgery rather than subjecting them to two laparoscopic surgeries simultaneously.

We have already stated in our methodology that this was a retrospective study and every retrospective analysis has its own limitations. Our numbers were small and therefore not matched for age and sex. The aim of our analysis was to highlight the advantages of concomitant laparoscopic surgeries.

Occasionally, we do combine endourological procedures with laparoscopic procedures; we have deliberately not analysed them in this article as this is an entirely different approach and it would have been difficult to compare keeping with the theme of the article. We hope to publish our experience with laparoscopic surgeries performed along with endourological procedures soon.

